# Attitude and compliance of dental assistants towards infection control: A quasi-experimental study

**DOI:** 10.12669/pjms.42.(11AASC).15604

**Published:** 2026-04

**Authors:** Faiza Ali, Rizwan Khalil, George Samuel, Rashna Hoshang Sukhia

**Affiliations:** 1 Dr. Faiza Ali, BDS, MSc, Masters in Digital Health and Data Science, University of Sydney, Australia; 2 Dr. Rizwan Khalil, BDS, Resident Orthodontics, Department of Dentistry & Oral Health Sciences, The Aga Khan University Hospital, Stadium Road, Karachi 74800, Pakistan; 3 George Samuel, Senior Dental Assistant, Department of Dentistry & Oral Health Sciences, The Aga Khan University Hospital, Stadium Road, Karachi 74800, Pakistan; 4 Dr. Rashna Hoshang Sukhia, BDS, MSc, FCPS. Consultant Orthodontist/Associate Professor, Program Director Orthodontics Residency Program, Department of Dentistry & Oral Health Sciences, The Aga Khan University Hospital, Stadium Road, Karachi 74800, Pakistan

**Keywords:** Compliance, Disinfection, Educational intervention, Environmental contamination, Hand hygiene, Infection control

## Abstract

**Objectives:**

The primary objective was to conduct a PDSA cycle on the infection control practices of dental assistants. The secondary objective of the study was to record the knowledge, attitudes, and perceptions of dental assistants regarding infection control practices.

**Methodology:**

The 15 months (December 2022- February 2024) quasi-experimental study followed a PDSA cycle with a sample size of 22 selected via non-probability purposive sampling. During the Planning Phase, a proforma was developed, and evaluators were trained for data collection. In the Do Phase, two evaluators collected data anonymously over six days for eight weeks. Analysis in the Study Phase revealed compliance rates below 95%, prompting an educator reinforcement session in the Act Phase to improve compliance. Paired pre–post analysis was conducted for assistants with complete data (n=18). The McNemar test was used to compare pre- and post-intervention compliance, while a self-structured questionnaire was shared via Google Forms for the secondary objective.

**Results:**

Twenty-two dental assistants were observed, with most infection control practices initially below 95%. After a reinforcement session, compliance improved, with significant gains in four practices. Sixteen participants achieved 100% compliance, and three showed statistically significant improvements (*p* = 0.002, 0.031, 0.003). Questionnaire responses showed 95.5% adherence to handwashing and infection control training, while 81.8% occasionally changed masks for new patients. Six assistants reported sharp injuries or exposure to body fluids in the past year.

**Conclusions:**

The implementation of the PDSA cycle proved to be highly effective in improving infection control compliance among dental assistants. The compliance improved to 100% after the administration of the educational reinforcement session.

## INTRODUCTION

Infection control and prevention of cross-contamination are essential for good quality healthcare services. Many micro-organisms, some of which are pathogenic, such as Herpes Simplex Virus, Varicella Zoster Virus, Human Immunodeficiency Virus, Hepatitis B, C, and D viruses, Mycobacterium spp., Pseudomanas spp., Legionella spp., and multi-resistant bacteria are found in the oral cavity. They can be a potential source of infection for other patients and dental healthcare professionals (DHCP).^1^,^2^ The transmission of pathogens can occur directly via blood, saliva, and droplet inhalation or indirectly via contaminated surfaces, light handles, triple syringes, etc.^3^,^4^ The Center of Disease Control and Prevention (CDC) formulated infection control policies for DHCP and dental clinics in 2003.^5^ These guidelines developed by CDC and other higher organizations include Standard Precautions (SP) that can be used to ensure a safe working environment.^6^

Infection control practices include hand hygiene, wearing personal protective equipment (PPE), sterilizing instruments, and disinfection of objects and surfaces. The staff should be regularly updated with the latest guidelines for infection control. The CDC also recommends up-to-date immunization status of the staff against hepatitis B and other pathogens.^7^

The Plan-do-study-act (PDSA) cycle, in healthcare settings, is utilized to optimize outcomes with iterative testing of changes to practices and continuous quality improvement. It can be used in dental settings for the identification and testing of infection control practices and interventions. The implementation of the PDSA cycle can improve compliance and efficiency in infection control protocols.^8^ Many countries have successfully integrated PDSA-driven interventions into their medical and dental infection control programs, demonstrating substantial improvements in compliance and patient safety.^9^,^10^ Infection control practices such as training of the staff, sterilization of equipment, and adherence to safety protocols have historically faced significant challenges in Pakistan.^11^ Although the situation is gradually improving, incorporating structured quality improvement processes like PDSA has been limited.

Anders PL et al.^12^, in their natural experiment to determine the adherence of dental students to infection control practices, reported that studies that depend on self-reporting are open to bias and that actual compliance is often overestimated. It is therefore important to assess compliance in real-life settings when the practitioners, assistants, and students are unaware of being assessed. The purpose of this quality improvement project was to conduct a PDSA cycle on the infection control practices of dental assistants in a tertiary care hospital.

## METHODS

The study was conducted using a Quasi-experimental study design using a PDSA cycle and the study duration was 15 months (December 2022- February 2024). A non-probability purposive sampling technique was used, and the sample size was 22. All dental assistants assigned to dental care clinics, at the Section of Dentistry, the Aga Khan University Hospital (AKUH) were included in the study. All those who did not consent to participate were excluded.

### Ethical Approval:

Approval from the Ethical Review Committee (ERC) of the Aga Khan University Hospital (AKUH) was obtained (ERC # 2023-8329-24116; dated March 2, 2023). The null hypothesis was that there would be no difference in the compliance scores of infection control practices among dental assistants after the PDSA cycle.

The study was conducted according to the four stages of the PDSA cycle. In the ‘Planning Phase, a proforma was developed and the evaluators were trained to collect the data to assess and record the infection control practices by the dental assistants before seating a new patient. In the ‘Do Phase’ the data was collected by the two evaluators who were not part of the study. The data was collected anonymously for six days for eight weeks (02/08/2023 to 02/10/2023). Some of the dental assistants were observed multiple times. In the ‘Study Phase’, the data was analyzed, and it was noted that the compliance level for all the practices was lower than 95%, therefore a reinforcement session was organized to improve the compliance rate among the dental assistants (‘Act Phase’). The session was organized as an interactive activity on Kahoot to improve the participation rate and to make it interesting. The participants were observed again for eight weeks after three months (05/12/2023- 02/02/2024) and the infection control practices were recorded.

For the secondary objective, a self-structured questionnaire was shared with the dental assistants via Google Forms. The questionnaire was developed based on the literature^4^,^6^,^13^ and had close-ended questions. We recorded their demographic data, hand hygiene practices, use of PPE, disinfection and sterilization, management of sharp waste material, immunization history, and training on infection control practices. Reminder emails were sent after every two weeks for two months to increase the response rate.

### Statistical analysis:

The recorded data from Google Forms was imported as a CSV file in MS Excel and later exported to SPSS Statistics version 23 (IBMSPSS Inc, Armonk, NY) for analysis. Percentage improvement has been reported pre and post-intervention. The Mc Nemar test was applied to compare each dental assistant’s compliance score. Knowledge, attitudes, and practices have been reported as frequencies and percentages.

## RESULTS

For the PDSA cycle, 22 dental assistants were observed for infection control practices for dental chair setup and procedures for two months. Each dental assistant was given a unique study ID. A total of 62 observations were recorded by the two evaluators. The results indicated that 29% of the time, the dental assistants did not wash their hands before chair setup and 17.7% of the time, they did not disinfect the unit using alcohol wipes. The plastic barriers were not changed from the unit (8.1%), tray (19.4%), tray handles (9.7%), and triple syringe (16.1%). Wraps were not changed from the unit light (16.1%) and key panel (11.3%). After the procedure was completed, 83.9% of the time the prime and bond bottle were not disinfected, 88.7% of the time the instrument was not disinfected before taking a composite increment, and the composite syringe was also not disinfected. 87.1% of the time the etchant syringe was not disinfected. The same practices were observed again after the reinforcement session (post-intervention) and improvement was seen on all the steps. A total of 79 observations were recorded this time. The highest percentage improvement was recorded for disinfecting the instrument after taking composite increment (86.2%) followed by disinfection of etchant and composite syringe (83.3% and 84.9% respectively). 96.2% of the time, the dental assistants washed their hands before the start of the procedure which was only 71% before the intervention (Table-I).

**Table-I:** Infection control practices observed pre and post-intervention

S. No.	Infection control practice	Pre-Intervention N (%)	Post-Intervention N (%)	Percentage Improvement
***Infection control measures before chair setup***				
	Handwash before chair setup			
	Yes	42 (67.7%)	76 (96.2%)	28.5%
	No	18 (29%)	-	
	Not Observed	2 (3.2%)	3 (3.8%)	
	***Unit Disinfection by Alcohol Wipes***			
	Yes	51 (82.3%)	79 (100%)	17.7%
	No	11 (17.7%)		
	***Change of Plastic Barriers from Unit***			
	Yes	57 (91.9%)	79 (100%)	8.1%
	No	5 (8.1%)		
	***Change of Plastic Barriers from the tray***			
	Yes	50 (80.6%)	65 (82.3%)	1.7%
	No	12 (19.4%)	14 (17.7%	
	***Change the Plastic barrier from the tray handle.***			
	Yes	56 (90.3%)	78 (98.7%)	8.4%
	No	6 (9.7%)	1 (1.3%)	
	***Change of all wraps from the unit light***			
	Yes	52 (83.9%)	79 (100%)	16.1%
	No	10 (16.1%)		
	Not Observed			
	***Change of all wraps from the key panel***			
	Yes	55 (88.7%)	79 (100%)	11.3%
	No	7 (11.3%)		
	Not Observed			
	***Change of Plastic Barriers from triple syringe***			
	Yes	52 (83.9%)	79 (100%)	16.1%
	No	10 (16.1%)		
	***Handwash after chair setup***			
	Yes	57 (91.9%)	79 (100%)	8.1%
	No	5 (8.1%)		
	***Handwash before procedure***			
	Yes	44 (71%)	76 (96.2%)	25.2%
	No	17 (27.4%)	1 (1.3%)	
	Not Observed	1 (1.6%)	2 (2.5%)	
	***Placement of new tip on etchant syringe***			
	Yes	60 (96.8%)	79 (100%)	3.2%
	No	2 (3.2%)		
	***Placement of barrier over light curing tip***			
	Yes	59 (95.2%)	79 (100%)	4.8%
	No	3 (4.8%)		
	***Placement of new tip of the triple syringe***			
	Yes	61 (98.4%)	79 (100%)	1.6%
	No	1 (1.6%)		
	***Disinfection of Prime bond bottle after use***			
	Yes	10 (16.1%)	78 (98.7%)	82.6%
	No	52 (83.9%)	1 (1.3%)	
	***Disinfection of instrument before taking composite increment***			
	Yes	7 (11.3%)	77 (97.5%)	86.2%
	No	55 (88.7%)	2 (2.5%)	
	***Disinfection of etchant syringe after use***			
	Yes	8 (12.9%)	76 (96.2%)	83.3%
	No	54 (87.1%)	3 (3.8%)	
	***Disinfection of Composite syringe after use***			
	Yes	7 (11.3%)	76 (96.2%)	84.9%
	No	55 (88.7%)	3 (3.8%)	
	***Handwash After Procedure***			
	Yes	58 (93.5%)	79 (100%)	6.5%
	No	3 (4.8%)	-	
	Not Observed	1 (1.6%)	-	

N = 22.

The compliance score of each participant before and after the intervention was also calculated and the McNemar test was applied to calculate the *p*-value. Although 22 dental assistants were observed overall, complete paired pre- and post-intervention data were available for 18 assistants, who were included in the McNemar (paired) analysis. The average compliance score became 100% for the majority of the participants (16 out of 18) and a statistically significant *p*-value was recorded for three of the dental assistants (0.002, 0.031, 0.004 respectively) (Table-II).

**Table-II:** Pre and post-intervention compliance score for individuals (Mc Nemar Test).

Study I.D.	Pre-intervention average compliance score (%)	Post-intervention average compliance score (%)	McNemar Exact Sig (2-sided)
1	14 (77.8%)	17 (94.4%)	0.375
2	14 (77.8%)	18 (100%)	0.125
3	15 (83.3%)	18 (100%)	0.250
4	13 (72.2%)	18 (100%)	0.63
5	8 (44.4%)	18 (100%)	0.002*
6	15 (83.3%)	17 (94.4%)	0.625
7	13 (72.2%)	18 (100%)	0.623
8	14 (77.8%)	18 (100%)	0.125
9	13 (72.2%)	18 (100%)	0.063
10	14 (77.8%)	18 (100%)	0.125
11	13 (72.2%)	18 (100%)	0.063
12	12 (66.7%)	18 (100%)	0.031*
13	18 (100%)	18 (100%)	-
14	14 (77.8%)	18 (100%)	0.125
15	14 (77.8%)	18 (100%)	0.125
16	9 (50%)	18 (100%)	0.004*
17	15 (83.3%)	18 (100%)	0.250
18	14 (77.8%)	18 (100%)	0.125

Only participants with complete paired pre- and post-intervention observations were included in McNemar analysis. N = 18, p ≤ 0.05*

The hand hygiene practices, use of personal protective equipment, disinfection, and sterilization practices were also recorded using a questionnaire. We received responses from 22 dental assistants to the questionnaire out of which 18 were males and four were females. The highest level of education was intermediate (college-level) for the majority of the participants (59.1%). The mean value for the length of practice was 11.6 with a standard deviation of 5.12 (Table-III). About 95.5% reported that they wash their hands before and after the patient treatment and that they have received infection control training. About 81.8% reported that they occasionally change masks for new patients while 36.4% reported that they occasionally change gloves between patients. Fifty percent of the assistants ask the patient to do pre-operative mouth rinses and 90.9% of the assistants reported that they immersed the used instruments in the decontamination solution and sterilized the handpiece. The results from these have been summarized in Table-IV.

**Table-III:** Demographic details of the participants.

Age	N (%)
19-30 years	3 (13.6%)
31 to 50 years	19 (86.4%)
***Gender***	
Males	18 (81.8%)
Females	4 (18.2%)
***Education***	
Intermediate	13 (59.1%)
Bachelors	5 (22.7%)
Diploma	4 (18.2%)

N = 22.

**Table-IV:** Infection control practices reported by the assistants (Questionnaire)

	Always	Occasionally	Never
Hand Hygiene Practices			
Washing hands before patient treatment	21 (95.5%)	1 (4.5%)	-
Washing hands after patient treatment	21 (95.5%)	1 (4.5%)	-
Washing hands before donning gloves	16 (72.7%)	6 (27.3%)	-
Using hand sanitizer instead of washing	8 (36.4%)	13 (59.1%)	1 (4.5%)
Use of Protective personal equipment			
Wearing gloves while performing dental procedures	(100%)	-	-
Changing gloves between patients	13 (59.1%)	8 (36.4%)	1 (4.5%)
Using sterile surgical gloves for surgery	10 (45.5%)	10 (45.5%)	2 (9.1%)
Wearing protective eyewear	16 (72.7%)	6 (27.3%)	-
Wearing mask	22 (100%)	-	-
Changing masks between patients	4 (18.2%)	18 (81.8%)	-
Wearing disposable gowns for surgery	14 (63.6%)	8 (36.4%)	-
Asking your patient to do preoperative mouth rinses	11 (50%)	10 (45.5%)	1 (4.5%)
Disinfection and sterilization			
Immersing used instruments in decontaminant solutions	20 (90.9%)	2 (9.1%)	-
Sterilizing of handpieces	20 (90.9%)	1 (4.5%)	1 (4.5%)
Sterilizing of burs	22 (100%)	-	-
Sterilizing of endodontic files	21 (95.5%)	-	1 (4.5%)
Use of wrapping bags for instrument sterilization	21 (95.5%)	1 (4.5%)	-
Use of surface barriers for dental unit surfaces	18 (81.8%)	4 (18.2%)	-
Use of routine wiping for surface disinfection	20 (90.9%)	2 (9.1%)	-
Chemical disinfection of impressions before sending to the laboratory	19 (86.4%)	2 (9.1%)	1 (4.5%)
Washing impressions with water before sending to the laboratory	20 (90.9%)	2 (9.1%)	-

N = 22.

About 95.5% reported that they have been vaccinated for the hepatitis-B virus and 77.3% have had a hepatitis B virus serology test done. Six dental assistants were exposed to sharp injuries or blood and body fluids in the last 12 months (Table-V).

**Table-V:** Sterilization, Management of sharp waste, Immunization history, and previous infection control training

Sterilization and Management of Sharps Waste		
Which of the following do you use to sterilize instruments in the dental clinic??	Autoclave	10 (45.5%)
	Washing	-
	Disinfectant Solution	-
	All of the above	12 (54.5%)
When do you immerse the used instruments in decontaminant solutions?	Before treatment 2 (9.1%)	After treatment 20 (90.9%)
	Yes	No
Do you have an appropriate protocol for emergency treatment of needle sticks or other sharp accidents?	22 (100%)	-
Exposed to sharp injuries or blood and body fluids in the last 12 months?	6 (27.3%)	16 (72.7%)
Hepatitis B virus vaccination	21 (95.5%)	1 (4.5%)
Have you ever had a Hepatitis B Virus serology test?	17 (77.3%)	5 (22.7%)
***Infection Control Training***		
Have received infection control training	21 (95.5%)	1 (4.5%)
Do you have written infection control guidelines in your department	22 (100%)	-
Received instructions about what to do in case of needlestick injury	22 (100%)	-

N = 22.

## DISCUSSION

The demographic data of dental assistants in this study revealed that the majority (86.4%) of participants were aged between 31–50 years. This finding aligns with a study conducted by Mutters et al.^2^, where a significant portion of participants in a university hospital dental clinic were middle-aged, suggesting that infection control responsibilities often fall to experienced staff members. Regarding gender, most participants (81.8%) were male. This contrasts with Farotimi et al.^14^, where the nursing workforce in Nigerian hospitals had a more balanced gender distribution. The higher proportion of males in our study reflects the gender dynamics in dental assistant roles at the study location.

In terms of education, 59.1% of the participants had completed an intermediate level of education. This is consistent with findings by Rajih et al.^15^, who reported that most ICU nursing staff in Iraq had moderate educational attainment. However, our study diverges from Gijare et al.^16^, which found higher education levels among healthcare professionals. The variation could be due to differences in role requirements and educational systems across regions. Concerning years of experience, 72.7% of participants had over 10 years of practice. This finding is similar to Marey et al.^17^, where long-tenured nursing staff demonstrated a strong presence in healthcare settings. The high experience level in our study may explain the initial partial compliance with infection control measures, as experienced staff might rely on routine rather than updated practices.

Before the intervention, compliance with infection control practices, such as hand hygiene and equipment disinfection, was suboptimal. For example, only 67.7% of participants washed their hands before chair setup. Following the educational session, compliance improved significantly, with handwashing increasing to 96.2%.

These findings align with Gaikwad et al.^18^, who reported improved compliance among nursing staff following educational interventions. Similarly, Ebrahiem et al. and Talaat et al.^19^ found that structured educational guidelines significantly enhanced adherence to standard precautions. The similarity likely stems from the universal effectiveness of interactive training in promoting behavior change across healthcare settings. However, our results differ slightly from Ahmad et al.^20^, where compliance rates for certain practices, such as equipment sterilization, improved less dramatically post-intervention. This discrepancy may be attributed to differences in intervention delivery methods, as our study incorporated gamification, which may have boosted engagement and retention of knowledge.

The questionnaire revealed that 95.5% of participants reported consistent handwashing before and after patient treatment, and 90.9% ensured proper sterilization of instruments. These findings are supported by Mahmoud et al.^21^, who observed significant improvements in infection control knowledge and practices post-training among nurses caring for critically ill patients. Interestingly, 36.4% of participants in our study occasionally changed gloves between patients, reflecting a gap in understanding best practices. This finding aligns with Marey et al.^17^, where some participants failed to adhere to glove-changing protocols. The persistence of this gap suggests that cultural or workload factors may influence adherence, which should be addressed in future interventions.

The compliance improvement observed in our study is comparable to findings by Mutters et al.^2^ and Gijare et al.^16^, where regular audits and targeted education significantly enhanced infection control adherence. These results collectively highlight the importance of combining observation, feedback, and education to drive sustainable behavior change. However, our study contrasts with Farotimi et al.^14^, where improvements were less pronounced. This difference might be attributed to variations in baseline knowledge, resource availability, or the intensity of interventions across study settings.

### Limitations:

It was conducted at a single institution with a small sample size, limiting the generalizability of the findings. The long-term retention of compliance improvements post-intervention was not assessed, and potential barriers, such as workload and resource availability, were not explored.

## CONCLUSIONS

The implementation of the PDSA cycle proved to be highly effective in improving infection control compliance among dental assistants. The compliance improved to 100% after the administration of the educational reinforcement session.

### Recommendations:

To address these limitations, future research should include larger, multi-center samples to enhance external validity. Long-term follow-up assessments could evaluate the sustainability of compliance improvements, and qualitative approaches, such as focus groups or interviews, could identify and address systemic barriers to adherence.

### Author`s Contribution:

**FA:** Conceptualization, Data curation, Formal analysis, Investigation, Methodology, Project administration. Accountable for the accuracy or integrity of the work

**RK:** Software; Writing – original draft, Writing - Review & Editing

**GS:** Data curation, Critical review of the manuscript

**RHS:** Supervision, Validation, Statistical analysis, Writing - Review & Editing.
